# CATH: increased structural coverage of functional space

**DOI:** 10.1093/nar/gkaa1079

**Published:** 2020-11-25

**Authors:** Ian Sillitoe, Nicola Bordin, Natalie Dawson, Vaishali P Waman, Paul Ashford, Harry M Scholes, Camilla S M Pang, Laurel Woodridge, Clemens Rauer, Neeladri Sen, Mahnaz Abbasian, Sean Le Cornu, Su Datt Lam, Karel Berka, Ivana Hutařová Varekova, Radka Svobodova, Jon Lees, Christine A Orengo

**Affiliations:** Institute of Structural and Molecular Biology, University College London, London WC1E 6BT, UK; Institute of Structural and Molecular Biology, University College London, London WC1E 6BT, UK; Institute of Structural and Molecular Biology, University College London, London WC1E 6BT, UK; Institute of Structural and Molecular Biology, University College London, London WC1E 6BT, UK; Institute of Structural and Molecular Biology, University College London, London WC1E 6BT, UK; Institute of Structural and Molecular Biology, University College London, London WC1E 6BT, UK; Institute of Structural and Molecular Biology, University College London, London WC1E 6BT, UK; Institute of Structural and Molecular Biology, University College London, London WC1E 6BT, UK; Institute of Structural and Molecular Biology, University College London, London WC1E 6BT, UK; Institute of Structural and Molecular Biology, University College London, London WC1E 6BT, UK; Institute of Structural and Molecular Biology, University College London, London WC1E 6BT, UK; Institute of Structural and Molecular Biology, University College London, London WC1E 6BT, UK; Department of Applied Physics, Faculty of Science and Technology, Universiti Kebangsaan Malaysia, Bangi, Selangor 43600, Malaysia; Regional Centre of Advanced Technologies and Materials, Department of Physical Chemistry, Faculty of Science, Palacký University Olomouc, Olomouc 771 46, Czech Republic; National Centre for Biomolecular Research, Faculty of Science, Masaryk University, Brno 602 00, Czech Republic; Central European Institute of Technology, Masaryk University, Brno 625 00, Czech Republic| National Centre for Biomolecular Research, Faculty of Science, Masaryk University, Brno 602 00, Czech Republic; Department of Biological and Medical Sciences, Faculty of Health and Life Sciences, Oxford Brookes University, Oxford OX3 0BP, UK; Institute of Structural and Molecular Biology, University College London, London WC1E 6BT, UK

## Abstract

CATH (https://www.cathdb.info) identifies domains in protein structures from wwPDB and classifies these into evolutionary superfamilies, thereby providing structural and functional annotations. There are two levels: CATH-B, a daily snapshot of the latest domain structures and superfamily assignments, and CATH+, with additional derived data, such as predicted sequence domains, and functionally coherent sequence subsets (Functional Families or FunFams). The latest CATH+ release, version 4.3, significantly increases coverage of structural and sequence data, with an addition of 65,351 fully-classified domains structures (+15%), providing 500 238 structural domains, and 151 million predicted sequence domains (+59%) assigned to 5481 superfamilies. The FunFam generation pipeline has been re-engineered to cope with the increased influx of data. Three times more sequences are captured in FunFams, with a concomitant increase in functional purity, information content and structural coverage. FunFam expansion increases the structural annotations provided for experimental GO terms (+59%). We also present CATH-FunVar web-pages displaying variations in protein sequences and their proximity to known or predicted functional sites. We present two case studies (1) putative cancer drivers and (2) SARS-CoV-2 proteins. Finally, we have improved links to and from CATH including SCOP, InterPro, Aquaria and 2DProt.

## INTRODUCTION

The CATH database, originally developed in 1997 (1), provides an up-to-date and systematic structural classification of protein 3D structures and is one of the Core Data Resources within ELIXIR, a major European distributed infrastructure for life-science information. CATH employs a semi-automated procedure to split 3D structures into their constituent domains (semi-independently folding globular units) and clusters these domains into homologous superfamilies where there is sufficient evidence of evolutionary ancestry (2,3).

In addition to classifying domains in PDB structures, CATH assigns domains for protein sequences for which 3D structures are unknown. As well as providing this data in CATH, we also provide the data in our sister resource, Gene3D (available at http://gene3d.biochem.ucl.ac.uk/Gene3D/ (4)). Both CATH and Gene3D provide comprehensive structural domain assignments and functional annotation for protein sequences from major protein sequence databases such as UniProt and Ensembl (5,6). To obtain this predicted domain data we use a set of representative structural domains to ‘seed’ a set of protein sequence alignments, which are converted into hidden Markov models (HMMs). HMMs are then used to identify closely related domains within protein sequences from UniProt and ENSEMBL. Thus, by combining protein structure and sequence, CATH provides comprehensive structure-based domain superfamily assignments for over 82 million protein sequences (151 million protein domains).

The domains are classified into the following hierarchical levels: Class (C), Architecture (A), Topology (T) and Homologous superfamilies (H) (1,3). For every superfamily, CATH provides structural superpositions of all representative protein domains using an in-house structure and sequence alignment program (SSAP) (7).

The members of Homologous superfamilies (H) share a conserved structural core, however in large superfamilies they often tend to have diverse functions. To address this, CATH has developed a functional classification protocol (FunFHMMer) utilising a hierarchical agglomerative clustering algorithm (8), to further sub-classify Homologous superfamilies (H) into functionally coherent groups known as Functional Families (referred to as FunFams). FunFHMMer segregates functional families on the basis of specificity-determining positions as well as highly conserved positions in cluster alignments and calculates a functional coherence index in order to determine functionally coherent alignments (8). For each FunFam, CATH provides sequence alignments (generated using MAFFT (9)), profile hidden Markov models (HMMs, generated using HMMER3 (10)), and a set of high-quality GO annotations from UniProt-GOA (11). As reported in the previous release, the CATH website provides a sequence-based search for identifying FunFams using query protein sequences (cathdb.info/search/by_sequence), or through the API.

FunFams tend to be more functionally coherent than other domain-based approaches (8), making them useful for predicting functional sites as well as protein structure. Function prediction pipelines developed using FunFams are consistently ranked among the top performers for Molecular Function and Biological Process Gene Ontology terms in the Critical Assessment of Functional Annotation competition (CAFA) (12,13).

Non-globular domains can cause problems during the initial domain chopping procedure. Since the release of CATH version 4.2, we have re-classified the non-globular superfamilies in a new Class 6 (6.x.x.x), separate from the main hierarchy. This special class now contains 790 superfamilies, and with continued curation efforts, we plan to include other special cases and architectures, such as short and synthetic peptides, fragments, linkers, nucleic acids and low resolution structures. A consequence of this reclassification brings down the number of SuperFamilies in the canonical 1–4 classification to a total of 5841.

The continuous deposition of structures and sequences in PDB and UniProt has led to significant expansions in the CATH superfamilies since the last release. Furthermore, superfamilies are unevenly populated and the 100 most populated CATH-Gene3D superfamilies contain around 54% of the >150 million sequences characterised in our resource. Among these, the top 11 ‘mega’ superfamilies contain millions of sequences, requiring novel approaches to reduce the computing time and processing power to properly classify them into functional families. Due to a newly redesigned functional classification pipeline, we can report an expansion of our functional families in CATH v4.3 to 212 872 families comprising 34 700 216 sequences, for which we can provide more accurate functional annotations. This article highlights improvements in our functional classification protocols, implemented to address the functional classification of superfamilies in general and of ‘mega-superfamilies’ in particular.

We also introduce new webpages displaying data generated by a new CATH-based protocol, CATH-FunVar (**Fun**ctional **Var**iation, https://funvar.cathdb.info/). This uses CATH-FunFams and the structural and functional annotations within them to highlight possible functional impacts of mutations in amino acid residues. To do this FunVar displays the proximity of residue mutations to known or predicted functional sites in the domain structure. We provide details of two initial use cases of FunVar applied to the analysis of genetic variations (namely residue mutations) in (i) putative cancer driver proteins (ii) SARS-CoV-2 proteins. The FunVar pages are generic and will be extended to display the structural location of variants in proteins from other important pathogens or other human proteins associated with disease.

## CATH v4.3 RELEASE HIGHLIGHTS

**Figure 1.**
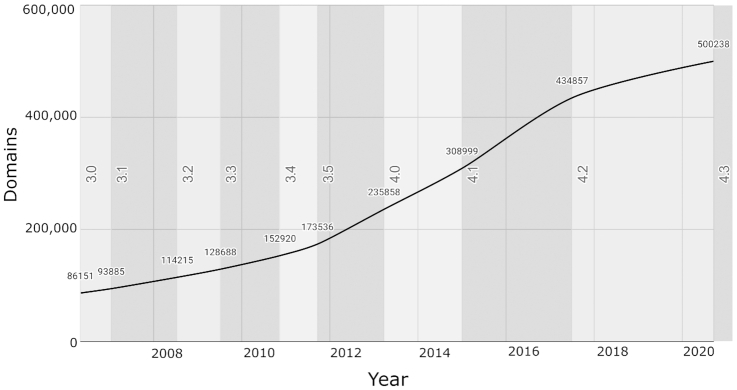
Number of structural domains classified in CATH releases over time.

The most recent CATH+ release, version 4.3 (based on PDB as of July 2019), brings a significant expansion in structural annotations (65 351 newly classified domains from 25 311 newly processed protein structures from the wwPDB); an increase of 15% since CATH+ release 4.2 (based on PDB as of July 2017) (Figure 1). The total number of superfamilies in the canonical classes 1–4 decreased from 6119 in the previous version to 5481 in the current version, due to the introduction of Class 6 for non-globular domains (which now contains 790 superfamilies). The corresponding sequence data for this release added an extra 56 million predicted domain sequences, a 59% increase.

The increase in sequence data, combined with a novel FunFam generation pipeline and curation efforts led to an overall increase in the number of FunFams from 68 065 to 212 872, covering an additional 1645 superfamilies (from 2683 in v4.2 to 4328 in v4.3, a 61% increase in coverage). However, this increase in coverage was not at the cost of purity, since the overall FunFam purity level and ‘Diversity of Positions Scores’ (DOPS) increased between the previous release and version 4.3 (see details below) (14).

Between v4.2 and v4.3, the CATH team have worked to improve the integration of CATH data with other resources, as well as tool developments for the general protein structure community. These efforts include new links to and from CATH, such as a new Domain Chopping platform developed with PDBe (EBI) and SCOP (UK) and CATH HMM model depositions in InterPro (EBI).

**Figure 2.**
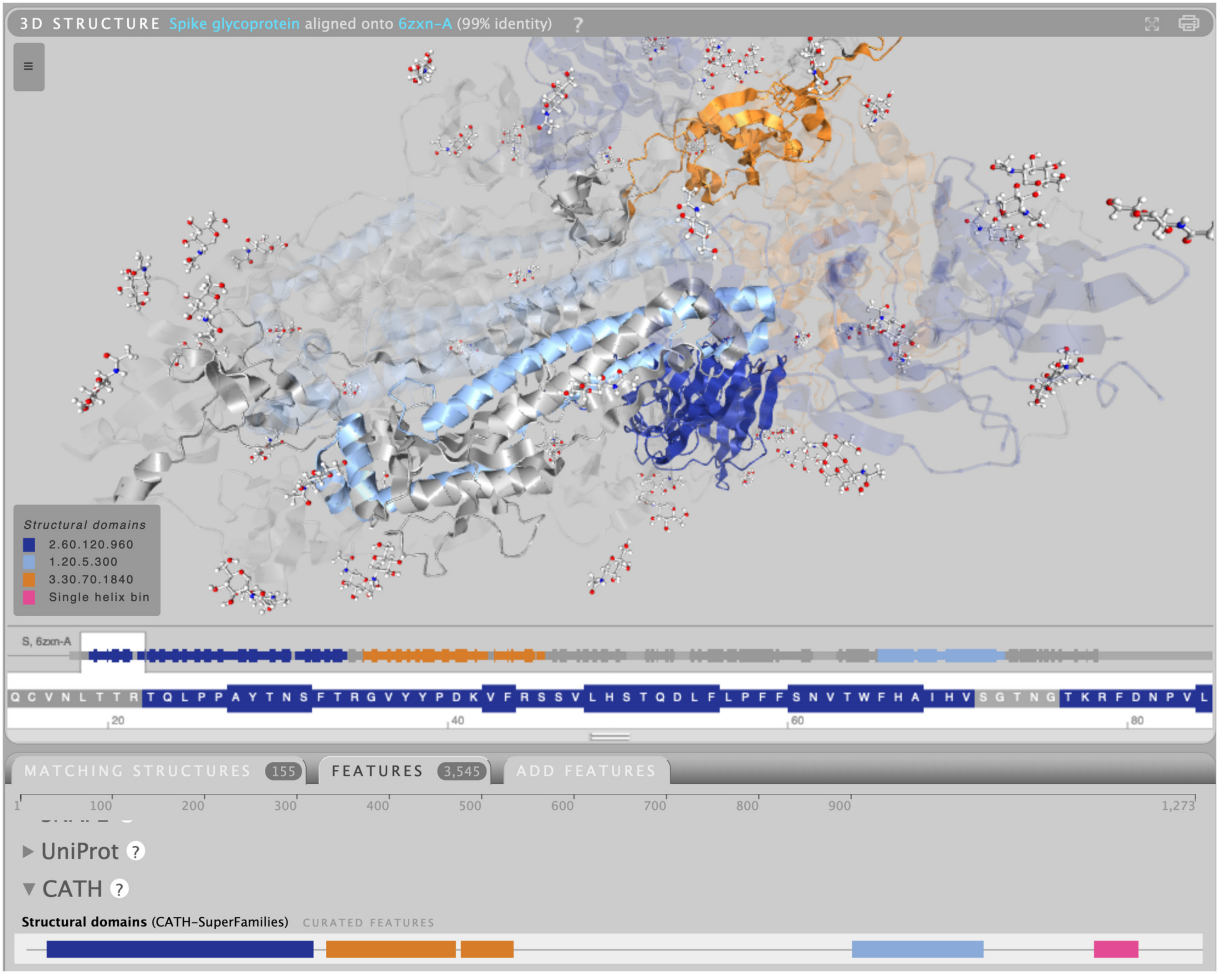
FunFam annotations of SARS-CoV-2 Spike protein as shown in Aquaria (https://aquaria.ws/P0DTC2/6zxn/A). Each FunFam domain in the sequence viewer matches the same domain in the 3D representation.

CATH features are now part of the comprehensive protein datasets displayed on Aquaria (https://aquaria.ws/ (15)), which was recently revamped to accommodate comprehensive information on SARS-CoV-2 proteins (16) (Figure 2). Descriptions, domain boundaries and various annotations (including GO, EC and taxonomic information) can now be visualized on the built-in structure visualizer. The Aquaria web server fetches the annotations programmatically from the CATH API as JSON files, while the user browser uses alternative endpoints to create compact, interactive data visualizations giving detailed information on the biological function and phylogenetic distribution of proteins containing a specific domain.

We have also included a novel visualisation of secondary structure arrangements in 2D space on our CATH SuperFamily Superposition pages (in collaboration with Radka Svobodova, CZ).

## ADDITIONAL FEATURES IN CATH

### Comprehensive and more accurate Functional Families (FunFams)

For the release of CATH v4.3, we devised a novel protocol for the generation of Functional Families (manuscript in preparation), allowing us to cope with the increase in sequence data whilst improving the processing time and functional purity. With this new protocol we have increased the number of FunFams from 68 065 to 212 872, a 3-fold increase (Table 1).

**Table 1.** Functional Families Statistics for CATH v4.3

FunFams Statistics			
Average Sequences per SuperFamily	7658	Sequences in High DOPS FunFams (≥70)	6 506 720
Sequences in FunFams	34 700 216	Sequences in low DOPS FunFams (<70)	1 940 795
CATH structural domains in FunFams	322 202	FunFams with DOPS>70	42 096
FunFams with CATH structure domains	17 208	FunFams with > = 5 sequences	96 249
% Gene3D in FunFams	35.65%	Filtered FunFams (DOPS>70, nseq >5)	39 540
Total number of FunFams	212 872	Sequences in FunFams / Total number of UniProt domains in Gene3D	36.5%
Number of sequences in FunFams (with structural representatives)/total number of UniProt domains in Gene3D.	5%	Structural Clusters (SSGs)	4990

Our approach pre-partitions the domain sequence data according to their predicted Multi-Domain-Architecture (MDA) context, based on the assumption that changes in a domain's context often drives changes in function (17). This strategy has been valuable as it allows us to process MDA partitions in parallel thereby reducing the processing time from six months (CATH v4.2) to six weeks (CATH v4.3) despite a significant increase in sequences classified in CATH superfamilies.

**Figure 3.**
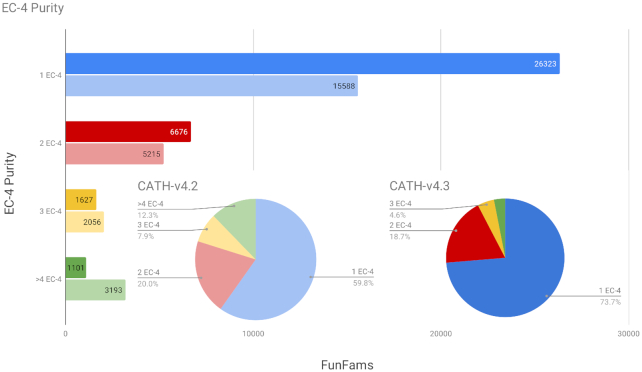
EC codes purity histograms for CATH 4.2 and 4.3 FunFams. The plot represents the number of FunFams with the same associated EC4 term across all sequences within the FunFam alignment. Only experimentally characterised EC terms were used in the validation. Pure FunFams have 1 EC4 term associated with them, two or more could be a potential indication of functional pollution. The overall EC purity in FunFams increased between releases.

This method has enabled us to process all the very large and diverse superfamilies (e.g P-loops, 3.40.50.300, over 9000 structural domains, 1.7 million domain sequences) from scratch, whilst for CATH version 4.2 we could only provide incremental updates. Furthermore, the speed improvements do not come at the cost of precision, since MDA partitioning has clearly improved the purity as judged by a benchmark based on experimental terms in the Enzyme Classification (EC) (Figure 3). The Enzyme Classification resource classifies enzyme function at 4 levels and provides a 4-number identification code for each classified enzyme. The first three numbers (EC3 in Figure 3) are assigned depending on the chemistry performed by the enzyme, whilst the fourth number (EC4 in Figure 3) reflects the substrate on which this chemistry is performed.

In addition, preliminary assessment in the CAFA4 critical independent assessment of functional annotation ranked our approach highly as with previous implementations (13). The new FunFams classification has increased the number of FunFams containing at least one structural representative, from 12 153 to 17 324 (+43%) and increased the number of unique GO terms captured within FunFams (+5%). A higher number of our FunFams have high information content as assessed by a DOPS score ≥70 (from 12 153 to 42 096) and therefore a deeper multiple sequence alignment for predicting functional sites (FunSites) and co-varying residues.

### 2DProts: visualising structural conservation within CATH superfamilies

**Figure 4.**
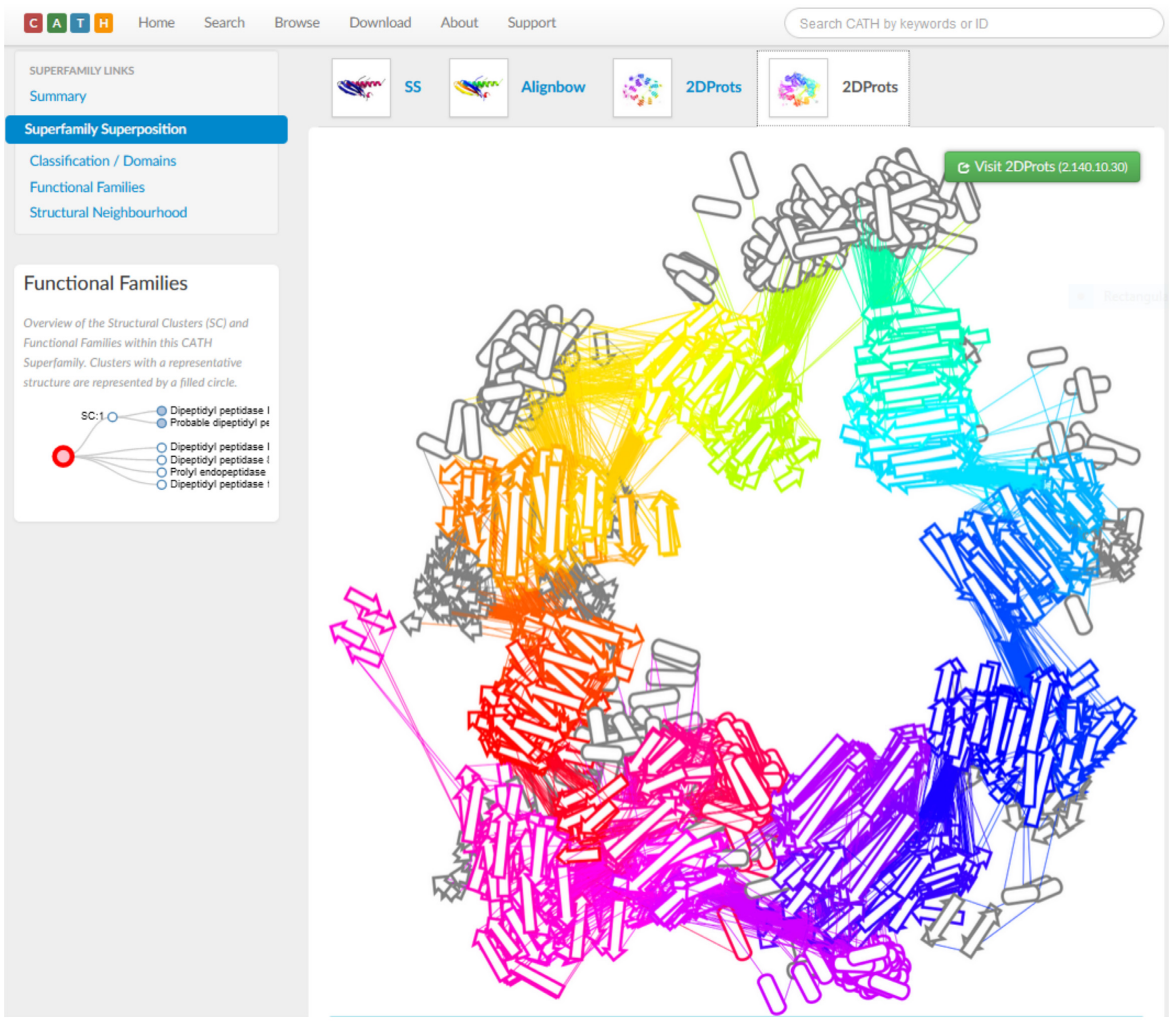
2DProts diagrams in the new CATH v4.3 pages provide a simplified view of the consensus topology for the domains within a given superfamily (SuperFamily 2.140.10.30, which adopts a beta propeller arrangement).

The 2DProts database (http://ncbr.muni.cz/2DProts) generates simplified secondary structure element (SSE) 2D diagrams for all entries from the PDB database and for the latest CATH domains and CATH superfamilies. These diagrams are created by deconstructing each CATH domain into constituent SSEs, then selecting the most highly conserved SSEs within the superfamily and placing those into the 2D diagram first. An evolutionary algorithm is used to calculate the relative position of all SSEs in order to reach as small a difference as possible between the 2D diagram and the original 3D structure. Hence, 2D diagrams correspond to all family members, i.e., chemically equivalent secondary structure elements can be found in the same place in all 2D diagrams of individual protein family members. The consensus patterns of superposed SSEs can provide a unique and clear overview of the conserved topology within the superfamily which can provide a valuable visualisation tool, especially for large superfamilies containing significant structural embellishments (Figure 4).

### CATH-FunVar

**Figure 5.**
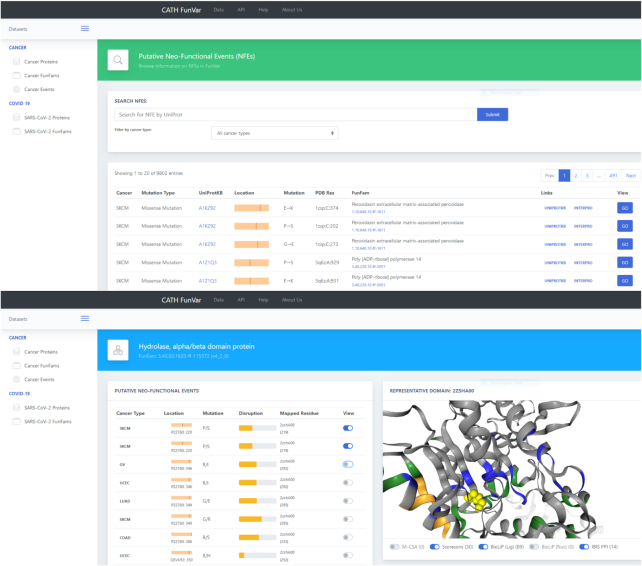
CATH FunVar web interface, highlighting all putative cancer mutations identified in CATH FunFams (top). On the bottom, we show mutations in one example FunFam. The left hand panel shows the degree of chemical change for each mutation, measured by the Grantham Score (25). Whilst the right hand panel shows a 3D representative, highlighting the locations of the mutations.

CATH-FunVar (**Fun**ctional **Var**iation) maps structural annotations, known and predicted functional sites and variants data (residue mutations) to FunFams, and is intended to showcase proteins with disease-associated variants and variants influencing host-pathogen interactions (Figure 5). The information integrated and derived by the FunVar platform is used to show whether residue variations lie within or in close proximity to interface regions and other functional sites, and could therefore have an impact on the protein function. The predicted functional sites shown by FunVar have been identified by detection of highly conserved residues in FunFam multiple sequence alignments (MSAs). We only predict putative functional sites for FunFams with highly informative MSAs (DOPS ≥ 70). Information content (DOPs) of the MSA and residue conservation score is determined using the scorecons algorithm (14).

### Data and platform design

We present two initial use cases to display the new FunVar webpages.

#### Human proteins associated with predicted cancer driver mutations

The FunVar pages display 2878 proteins which have predicted cancer driver mutations likely to have an impact on the protein function. Predicted driver mutations were identified by determining whether they lie within 3D clusters in the protein structure, enriched with predicted driver mutations (MutClusts). MutClusts were identified using our in-house protocol (18) and previously applied to identify putative cancer driver genes in 32 different cancer types. The predicted driver mutations displayed by FunVar also lie in or near a known or predicted functional site and may therefore have an impact on function. The FunVar web-pages display an image of the domain structure with the putative driver mutation and functional sites highlighted. These annotations either report experimental characterisation of the protein or inheritance of an experimental annotation across the FunFam.

#### Variations in a viral pathogen

As an initial case study we have presented data integrated for FunFams associated with COVID-19, i.e. FunFams containing SARS-CoV-2 proteins. UniProt hosts 14 SARS-CoV-2 proteins, catalogued as either individual proteins or polyproteins before cleavage. The entries were mapped to CATH FunFams using the 4.3 FunFams HMM library and cath-resolve-hits (19). We assigned two SARS-CoV-2 polyproteins, NSP7 and the spike glycoprotein to 26 different FunFams.

For each viral protein we obtained information on multiple strains (for SARS-CoV-2) from GISAID (20,21) and identified variants at each position in the sequence. As with the putative cancer mutations, the FunVar web-pages show viral or host protein mutations on a representative structure for the FunFam, with any known or predicted functional sites highlighted on the structure as well.

In summary our new FunVar web-pages allow the user to view the location of any residue mutations on the proteins structure and inspect their proximity to known or predicted functionals sites to assess the likely impact on protein function. They will also provide links to CATH pages where we show known or predicted EC terms and GO functional annotations from the FunFam in which the protein has been classified. In the future these pages will be extended to provide FunVar pages for the putative human interactors of SARS-CoV-2 identified by Gordon *et al.* using affinity-purification mass spectrometry (22).

Future releases will also provide additional information on drug compounds associated with relatives in the FunFam and other functional annotations e.g. from KEGG and REACTOME.

## CONCLUSIONS

Our new release of CATH represents a significant expansion in both structure (15% increase) and sequence (59% increase). In addition, we have improved the accuracy of our functional family classification and increased the number of functional families, representing a 59% increase in the structural coverage of GO functional space.

The inclusion of genetic variant data for proteins classified in CATH-FunFams allows us to display residue mutations on a structural representative for the functional family in which the variant protein has been classified, highlighting the proximity of the known mutation or predicted functional sites. In future the addition of drug target data to the FunVar web-pages will be useful in suggesting compounds that could be used to target the variant protein. Information on likely impacts of mutations can be valuable in the context of drug design and resistance, as well as disease severity.

The CATH-FunVar platform will also be used for providing functional annotation data for other pathogens and human diseases, such as tuberculosis. Our group has previously exploited the FunVar protocol to identify putative driver genes in a number of cancer types, i.e. by recognising variants that accumulate on or close to known or predicted functional sites (23). We have also recently used our FunVar platform (utilising FunFams) to study the impact of variants on the interaction of the SARS-CoV-2 spike with a range of different animal ACE2 proteins in order to understand host susceptibility to a broad range of animals (24).

In the future, we plan to integrate mutation/variation data from other resources (such as CoV-Glue, COVID-19 BEACON for SARS-CoV-2) and variations in human proteins (e.g. the UK BioBank) to better characterise the possible impacts on SARS-CoV-2 interactions and suggest potential targets to aid in therapeutics.

## DATA AVAILABILITY

The CATH website is available at: https://www.cathdb.info/; CATH FunVar is available at: https://funvar.cathdb.info. All the data is freely available and can be downloaded from the CATH website at https://www.cathdb.info/wiki?id=data:index.
